# Embracing Connection: A Review of First-Ever Clinical Guidelines on Social Isolation and Loneliness in Older Adults

**DOI:** 10.3390/geriatrics9050117

**Published:** 2024-09-11

**Authors:** Peter M. Hoang, David Conn

**Affiliations:** 1 Department of Medicine, Division of Geriatric Medicine, University of Toronto, Toronto, ON M5S 3H2, Canada; peter.hoang@medportal.ca; 2 Baycrest Health Sciences, North York, ON M6A 2E1, Canada; 3 Department of Psychiatry, University of Toronto, Toronto, ON M5T 1R8, Canada

**Keywords:** loneliness, social isolation, clinical practice guidelines, older adults

## Abstract

Social isolation and loneliness are major public health concerns and are associated with morbidity and mortality. As this is an increasing issue in older adults, guidance for healthcare providers is a priority. The Canadian Coalition for Senior’s Mental Health (CCSMH) has developed the first Canadian social isolation and loneliness guidelines to help providers recognize, assess, and manage social isolation and loneliness among older adults. We review and summarize these guidelines to support healthcare and social service providers to apply best practices and evidence-based care for older adults experiencing social isolation and loneliness.

## 1. Introduction

Social isolation and loneliness have major public health implications and remain a challenge internationally [1,2]. Loneliness is defined as the subjective feeling of unmet social needs or the feeling of being lonely [3]. Social isolation is defined as few or infrequent social contacts or the objective lack of social contact with other individuals [3]. Older adults (generally defined as 65 years of age or greater) [4] are particularly vulnerable to loneliness and social isolation due to changes in social structures, medical comorbidities, and living settings [5,6,7,8]. Given that over 10% of the global population is aged 65 years and over [9,10], social isolation and loneliness have far-reaching impacts and consequences for older adults [11]. Further, up to 58% of Canadian adults over fifty years of age experience loneliness, and 41% are at risk of social isolation [12]. European and American prevalence estimates of loneliness have reported that this issue affects up to one third of older adults [3,13,14]. As best practices in prevention, assessment, and management of social isolation and loneliness are paramount for healthcare and social service providers, the Canadian Coalition for Senior’s Mental Health (CCSMH) published the Canadian Clinical Guidelines on Social Isolation and Loneliness in Older Adults in 2024 [15]. We describe the development of these guidelines as the first clinical practice guidelines on this issue and highlight key practice points to inform healthcare providers. 

## 2. Development of the Practice Guidelines

The Canadian Coalition for Senior’s Mental Health is a not-for-profit interprofessional organization that was established in 2002 with the mandate of improving the mental health of older adults. Since then, the CCSMH has published clinical guidelines on mental health in long-term care, suicide prevention, depression, delirium, substance use disorders, behavioral and psychological symptoms of dementia, and anxiety. 

The CCSMH created a Guideline Development Working Group of nine core members (including the authors of this commentary) with diversity in gender, clinical practice/expertise, professional discipline, and Canadian geographical area. These included one geriatric psychiatrist, one geriatrician, one care of the elderly family physician, two social workers, one occupational therapist, and two researchers. The CCSMH completed a rapid scoping review of reviews and identification of gray literature [16]. This was followed by a national survey to capture perspectives from healthcare providers and older adults. Due to the opportunity for many different healthcare professionals to identify and support older adults experiencing social isolation and loneliness, our aim was to provide guidance across multiple disciplines. As such, we use healthcare and social service providers (HCSSP) to broadly include all professionals who provide care for older adults. 

The working group divided the sections by area of expertise for guideline drafting, later reaching consensus during teleconference meetings and subsequently voting on the guidelines. This methodology has been used in previous guidelines published by the CCSMH [17,18,19]. Sections of the guidelines were divided into Prevention, Screening and Assessment, and Interventions. These provide a framework for the healthcare provider across the spectrum from health promotion to management [20]. Interventions were divided by their primary intended category based on previous research [21,22]. The working group applied a modified version of the Grading of Recommendations, Assessment, Development, and Evaluation (GRADE) to assess the quality of the evidence (low, moderate, or high) for each recommendation and its available strength (weak or strong) [23]. In brief, the GRADE domains assess the risk of bias, imprecision, inconsistency, indirectness, and publication bias to rate the confidence in the available literature [23]. The modified approach further incorporates the confidence that patients will benefit from the action. Recommendations are then rated up or down, respectively, to obtain a scale from low to high. High certainty recommendations suggest a high degree of confidence in the true effect, and the opposite for low certainty recommendations. 

The strength of the recommendation indicates the following: (1) the degree of confidence in which the proposed action (e.g., prevention, assessment, or intervention) has desirable effects that outweigh negative consequences; (2) uncertainty or variability in a patient’s values and preferences; and (3) the resources associated with the action. The balance of these factors are used to designate the strength of the recommendation (weak or strong). Recommendations that had limited evidence but were considered best clinical practice were categorized as consensus recommendations. Assessment criteria for the quality of the evidence and the strength of the recommendations can be found in Table 1 and Table 2, respectively. All recommendations were independently voted on by committee members and were adopted if they had at least 75% approval; consensus was reached on all recommendations. These guidelines were reviewed by three external academic experts to provide feedback prior to publication. 

## 3. Applying the Guidelines to Clinical Practice 

These guidelines are divided into sections on Prevention, Screening and Assessment, and Interventions (Table 3) and are briefly summarized below. We recommend that healthcare providers apply these guidelines to all practice settings. This may include primary care offices and clinics, hospitals, and community and government agencies, amongst others. 

### 3.1. Prevention (Recommendations 1–3)

Healthcare providers should have knowledge of the risk factors associated with social isolation and loneliness in older adults, and these should be a core part of the educational curriculum. These risk factors include, but are not limited to: advanced age [24,25], female sex [26,27], identifying as 2SLGBTQIA+ (2S: Two-Spirit; L: Lesbian; G: Gay; B: Bisexual; T: Transgender; Q: Queer; I: Intersex; A: Asexual, and + [inclusive of people who identify as part of sexual and gender diverse communities]) [24,25,28], identifying as an ethnic minority [29], living alone, episodic mental or physical health issues [26], and being a caregiver/care partner [30]. Further, as healthcare and social service providers are an important point of contact for older adults, they should leverage their role and knowledge to educate patients and the public about social isolation and loneliness. 

### 3.2. Screening and Assessment (Recommendations 4–7)

The CCSMH recommends targeted screening for those who have risk factors using evidence-based screening tools. These can include single-item measures [31], the UCLA Loneliness Scale [32], and the Lubben Social Isolation Scale [33], among others. Care providers should recognize that each tool may not capture loneliness and isolation in its entirety, as tools can capture components of each based on the degree of subjectivity and the degree of structure in the social relationships [3]. In those who have been identified as having social isolation and/or loneliness, a thorough review of the medical and social history, precipitating factors (e.g., life events), psychiatric symptoms, insight, and motivation for change should be prompted. When social isolation and loneliness are detected, these should be documented in health records as a social determinant of health. 

### 3.3. Interventions (Recommendations 8–17) 

Care providers should ensure that alternative etiologies are initially or concurrently managed (e.g., medical or mental health conditions). It is important that healthcare providers take an individualized approach with shared decision-making to identify interventions that balance the individual’s goals and preferences with the available evidence and local resources. Interventions may include social prescribing, physical activity, psychological therapies (e.g., cognitive behavioral therapy), leisure skill development, animal therapy, and technology [21]. While these are highlighted as potential management strategies, there remains an important research gap in implementation studies, cost-effectiveness, and the duration of the effect. 

### 3.4. Special Populations

The guideline also considers the diversity of older adults and their personal experiences. As described in the *Interventions* section, tailored interventions should account for an individual’s sex, gender, culture, and personal identity. As such, this section highlights the importance of considering 2SLGBTQIA+ communities [12], Indigenous status [34], individuals who are refugees or immigrants [35], those living in long-term care [36], and dementia [37] when supporting those experiencing social isolation and loneliness. This is an area that requires further research. 

## 4. Contextualizing the Guidelines in the Current Landscape

In 2011, the United Kingdom launched the Campaign to End Loneliness. Over a decade later, it created a legacy as one of the first multidisciplinary hubs on the topic, with far-reaching influences [38]. In 2020, the World Health Organization endorsed the United Nations Decade of Healthy Ageing [39]. That same year, the National Academies of Science, Engineering, and Medicine released a consensus report on social isolation and loneliness [3]. Since then, there have been national reports, including the U.S. Surgeon General’s Advisory, calling for a national strategy to improve health and social systems [40]. Australia’s report on “Ending Loneliness Together in Australia” made similar recommendations and calls to action for a national plan [41]. The CCSMH guidelines echo similar national calls to action, in addition to providing practical tools that can be used in a point-of-care setting for the clinician and healthcare service provider. These guidelines can also be used to inform researchers and policymakers of best practices and future areas of study. While many studies have shown the association of social isolation and loneliness on negative clinical outcomes [11,42,43], there is a gap in identifying whether interventions that reduce social isolation and loneliness can improve clinical outcomes. 

## 5. Future Directions

Healthcare and social service providers routinely witness the inequities implicit in the social determinants of health [44]. Social isolation and loneliness are closely tied to these and may be important in the causal pathway to negative clinical outcomes [45,46]. Further research is required to understand the experiences of high-risk groups, consistency in the findings from interventions, implementation strategies, and the associations to clinical outcomes upon prevention and management. Policymakers, healthcare professionals, and researchers must remain connected to the needs of the population, identifying further areas for research and health policy. 

## 6. Conclusions

These guidelines apply the current evidence to support healthcare and social service providers and should be used as a tool to prevent, assess, and manage social isolation and loneliness. It should also be used by researchers and policymakers to guide future research and best practices. 

## Figures and Tables

**Table 1:** Summary of the assessment criteria used to determine the quality of evidence.

Quality of the Evidence	Description
High	Further research is unlikely to change confidence in the estimate of effect.
Moderate	Further research is likely to have an important impact on the confidence in the estimate of effect and may change the estimate.
Low	Further research is very likely to have an important impact on the confidence in the estimate of effect and is likely to change the estimate.

Adapted from the CCSMH Clinical Practice Guidelines for Social Isolation and Loneliness.

**Table 2:** Summary of the assessment criteria used to determine the strength of the recommendation.

Strength of the Recommendation	Description
Strong	Strong recommendations indicate high confidence that the desirable consequences of the proposed course of action outweigh the undesirable consequences or vice versa. In some cases, strong recommendations are made without high-quality evidence.
Weak	Weak recommendations indicate that there is either a close balance between benefits and downsides (including adverse effects and burden of treatment), uncertainty regarding the magnitude of benefits and downsides, uncertainty or great variability in patients’/clients’ values and preferences, or that the cost or burden of the proposed intervention may not be justified.

Adapted from the CCSMH Clinical Practice Guidelines for Social Isolation and Loneliness.

**Table 3:** Summary of recommendations for Prevention, Screening and Assessment, and Interventions for social isolation and loneliness.

Recommendation	Evidence	Strength
Prevention		
1. Healthcare providers should have knowledge of risk factors for social isolation and loneliness in older adults.	Moderate	Strong
2. Education and training about loneliness and social isolation should be a part of the curriculum for healthcare and social service professionals.	Consensus	
3. Healthcare and social service professionals should use their role, as agents of change, to help inform and educate patients/clients and the general public about the association between social isolation and loneliness and poor mental and physical health, and to promote social connection.	Consensus	
Assessment and Screening Tools		
4. HCSSPs should use targeted screening for those older adults who have risk factors for social isolation and loneliness.	Consensus	
5. When screening patients/clients, HCSSPs should use evidence-based screening tools to identify patients/clients who are socially isolated and/or lonely, to assess the severity of the problem, and in routine follow-up to determine whether the patient’s/client’s social situation has changed and whether interventions are effective.	Moderate	Strong
6. When social isolation and loneliness are identified in older adults, they should be documented in the health record like other medical conditions and risk factors. Efforts should be made to collect data on social isolation and loneliness as important social determinants of health. Loneliness and social isolation may be considered “psychosocial vital signs” given their impact on health.	Consensus	
7. A thorough clinical assessment with a patient/client who is socially isolated and/or lonely should aim to explore the possible causes and identify any underlying health conditions that may be contributing factors. Other causes that may be contributing should also be identified, adopting a biopsychosocial approach. A comprehensive assessment can guide the development of an appropriate management plan. The assessment may vary according to the healthcare and social service professional’s scope of practice.	Consensus	
Interventions		
8. HCSSPs should apply several principles to help older patients/clients who are socially isolated and/or lonely, including: Ensure initially or concurrently that treatment is provided for any underlying medical conditions identified in their assessment. Take an individualized approach with shared decision-making. Identify individuals’ interests to determine interventions that may be the best fit, while appraising the individual and environmental resources available. Recognize the diversity within older adult populations and, together with their patient/client, consider the incorporation of their culture and lived experience.	Consensus	
9. Social prescribing Social prescribing should be considered to manage or alleviate social isolation and loneliness. This can include connecting individuals with suitable organizations, programming, or community resources. Link workers and system navigators can support the assessment of an individual’s needs and connect them with suitable organizations. Community organizations and healthcare providers should collaborate and build relationships to support implementation.	Moderate	Strong
10. Social activity HCSSPs should support, encourage, and empower individuals to engage at their optimal level of social activity.	Moderate	Strong
11. Physical activity HCSSPs should encourage their patients/clients to engage in group and/or individual physical activity as a means to reduce social isolation and loneliness and to improve their overall health.	Moderate	Strong
12. Psychological therapies Psychological therapies include, but are not limited to, cognitive behavioral therapy, social cognitive therapy, reminiscence therapy, and mindfulness-based stress reduction. There is greater available evidence for psychological therapies in reducing loneliness compared to social isolation.	Moderate	Strong
13. Animal-assisted therapies and animal ownership Animal-assisted interventions and pet ownership may be helpful to some individuals although the evidence for this intervention is limited.	Low	Strong
14. Leisure skill development and leisure activities Healthcare providers should discuss leisure skill development and activities. This can include leisure education, art therapy, horticulture, and music therapy, amongst others.	Low	Weak
15. Technology Healthcare providers should engage patients and consider technology as a potential opportunity. This may include teleconferencing, use of the Internet/social media, and conversational agents.	Moderate	Strong
16. HCSSPs should not use pharmacological agents as treatments for social isolation and loneliness in older adults. Due to the lack of generalizable studies in humans and the potential for harm, we recommend against pharmacotherapy.	Low	Strong
17. HCSSPs should take an individualized approach to the follow-up of social isolation and loneliness. Due to the limited studies on serial reassessment and lack of evidence on effect sustainability, an individualized, short-term follow-up is recommended.	Consensus	

Adapted from the CCSMH Clinical Practice Guidelines for Social Isolation and Loneliness.

## Data Availability

The original contributions presented in the study are included in the article/supplementary material, further inquiries can be directed to the corresponding author/s.

## Supplementary Materials

The guidelines can be found at the following link: https://ccsmh.ca/areas-of-focus/social-isolation-and-loneliness/clinical-guidelines/ [47]. Further resources include: (1) For healthcare providers: https://ccsmh.ca/areas-of-focus/social-isolation-and-loneliness/health-care-professionals/ (accessed on 1 September 2024); (2) For older adults (includes information brochures): https://ccsmh.ca/areas-of-focus/social-isolation-and-loneliness/older-adults-and-care-partners/ (accessed on 1 September 2024); (3) Summary of our key findings, including the survey of health and social service providers and older adults: https://ccsmh.ca/areas-of-focus/social-isolation-and-loneliness/key-findings/ (accessed on 1 September 2024).
